# Factors Affecting Hypertensive Treatment Adherence: Development and Preliminary Validation of a New Scale

**DOI:** 10.1155/2021/6617980

**Published:** 2021-03-18

**Authors:** Mahlagha Dehghan, Nahid Dehghan Nayeri

**Affiliations:** ^1^Nursing Research Center, Kerman University of Medical Sciences, Kerman, Iran; ^2^Nursing and Midwifery Care Research Center, School of Nursing and Midwifery, Tehran University of Medical Sciences, Tehran, Iran

## Abstract

**Design:**

A sequential exploratory mixed method study was used.

**Methods:**

After item generation using a qualitative study and literature review, the psychometric properties of the scale were evaluated. Face, content, and construct validity, Cronbach's alpha, and test-retest reliability were used to validate the scales.

**Results:**

Data analysis showed that the scale had acceptable face and content validity. The scale had excellent stability (intraclass correlation = 0.89) and good acceptability of internal consistency (*α* = 0.71). The exploratory factor analysis showed that the scale consisted of five subscales which were meaningful.

**Conclusion:**

Psychometric properties of the scale achieved the standard level, and it was sufficient to recommend for general use in future measures of caring in nursing.

## 1. Introduction

Hypertension is one of the most prevalent factors that results in myocardium infarct, brain events, cardiac and renal failures, and early death [1]. Evidences suggest that prevalence of hypertension is increasing and sudden death resulting from hypertension has been reported in countries such as China, India, Iran, Sri Lanka, Pakistan, and Philippine [2, 3]. Haghdoust and Sadeghi Rad [4] showed in their meta-analysis that prevalence of hypertension in Iran is 23% at ages between 30 and 55 and 50% at ages older than 55 years [5].

One of factors involving in this situation is inappropriate control of hypertension in affected people [6]. According to report of the World Health Organization, more than half of patients whose hypertension is being treated do not continue treatment in the first year of diagnosis and half of those who continue their treatment take only 80% of prescribed medications. Therefore, hypertension of 75% of patients is not well controlled due to poor adherence to treatment regime [7]. In the study with approach of ground theory about hypertension control in Iran, Mohammadi et al. concluded that one of factors of lack of hypertension control in Iranian patients is that the patient does not adhere to his/her treatment regime [8]. Therefore, lack of adherence to treatment regime is considered as one of important clinical problems in treatment and control of chronic diseases and it can be followed by increasing costs of care, hospitalization rate, and increase of early death [9].

One of the ways of identifying causes of nonadherence is to explore perception of patients affected by hypertension from their diseases. In the studies with aim of exploring perception and view of patients affected by hypertension, Jolles et al. extracted three items including knowledge of hypertension, hypertension control day by day, and personal feelings and beliefs of the patient about being well. They found that knowledge may not be effective on the adherence, but it may be effective when knowledge is combined with factors related to the patient such as personal beliefs. Also, lack of a regular program for using the medication is the main obstacle reported for hypertension control. This study also indicated that feelings and beliefs of the patient about being well can be effective on adherence to medications prescribed by doctor and changes of life style and some people who believe that hypertension causes physical complications do not follow treatment measures until they feel well [10]. Systematic review of qualitative studies (America, Britain, Brazil, Swede, Canada, Denmark, Finland, Ghana, Iran, Israel, Netherland, southern Korea, Spain, Tanzania, and Thailand) on perception of hypertensive patients from their diseases and medicinal adherence indicated that many participants thought that hypertension is basically created by stress and causes symptoms such as headache, dizziness, and sweat. Participants stopped or reduced their treatment deliberately without consulting with the doctor. Most of participants believed that when symptoms were stopped or when they were not under stress, their blood pressure was better. As a result, they did not require treatment. Participants did not like its treatment and side effects, and they did not want to become dependent on it. Such findings were similar in different racial groups and cultures [11]. It seems that adherence to treatment regime has a low relationship with demographic and social factors such as age, gender, race, intelligence, and education. Waiting a long time for visiting the doctor in the clinic or long intervals between visits causes that patients do not continue treatment and visit the doctor. As a result, adherence to treatment regime reduces with increase of complexity, cost, and length of treatment regime [12].

Regarding the measuring tool of factors affecting treatment adherence in hypertensive patients, review of literature studies showed that there is no such a tool. According to conceptual framework offered by the World Health Organization in 2003, all factors affecting adherence of patients to chronic diseases are classified in five classes or dimensions. These dimensions are factors related to the patient, factors related to the treatment, factors related to treatment system, socioeconomic factors, and factors related to disease [13]. Although Ma et al. recommended in their study that such conceptual framework can make nonadherence possible [14], concerning literature review, this conceptual framework has not yet been considered in methodological studies as a conceptual framework for factors affecting treatment adherence. Two tools of “belief in medication” and “perception of disease” are the most applicable tools available for factors affecting treatment adherence [15, 16]. It is clear that questionnaire of belief in medication only concentrates on the belief and attitude of patient towards medications in general and medications taken for the disease in particular [15, 17]. The questionnaire of disease perception focuses on different dimensions of patient perception from his/her disease [16]. Therefore, the tools related to factors affecting the adherence are not sufficiently comprehensive and measure only some of causes effective on treatment adherence. Also, such tools do not concentrate on a special disease. It seems that there is no tool that could evaluate completely factors affecting treatment adherence of hypertensive patients [18]. Therefore, the present study aims to design and validate the tool “factors affecting treatment adherence in hypertensive patients.”

## 2. Methods and Materials

### 2.1. Study Design

This was a sequential exploratory mixed method study conducted in Kerman University of Medical Science and its affiliated educational hospitals and physician offices. Kerman is the largest city in southeastern Iran with a population of 722,000.

### 2.2. Item Generation

Development of the factors affecting hypertension treatment adherence (FEHTA) scale was guided by two key principles: (1) comprehensiveness, the scale was required to be comprehensive enough that it could consist of all factors affecting on hypertensive treatment adherence, and (2) universality, the scale was intended to capture the concept of FEHTA broadly so that it could be administered in other Iranian and non-Iranian clinical settings. Therefore, to capture these principles, both a qualitative content analysis and literature review were conducted to generate the item pool.

### 2.3. Qualitative Content Analysis

A qualitative content analysis was conducted to explore experiences of hypertensive patients (*n* = 10), their families (*n* = 4), and healthcare providers (*n* = 4) about factors affecting on treatment adherence in case of hypertension. The result of this study has reported elsewhere with details [19]. According to the results, a 311-item pool was developed from participants' quotations. In several meetings in the research team, some items that were similar and redundant or had overlapped were omitted or integrated to other items. Finally, at this phase, the scale consists of 82 items. These items were categorized into 4 conceptual dimensions including (1) personal factors (50 items), (2) disease and treatment nature (10 items), (3) health system role (9 items), and (4) family-socio-cultural factors (13 items).

### 2.4. Literature Review

To enrich the scale items and to ensure that all affecting factors were considered, the literature was reviewed. The databases of PubMed, ScienceDirect, Ovid, and Google Scholar were searched using the terms “treatment adherence,” “treatment compliance,” “treatment concordance,” “medication adherence,” “medication compliance,” “medication concordance,” “hypertension or high blood pressure,” “questionnaire,” “scale,” “instrument,” and “psychometric property,” “test-retest reliability,” “internal consistency,” “Cranach's alpha,” “construct validity,” “content validity,” “face validity,” “guidelines,” “content analysis,” and “qualitative study.” The search was limited to electronic English articles, and no publication date restrictions were applied. Totally, 147 articles were retrieved. Among these, the most relevant articles that could help us to enrich the pool items were selected. Therefore, three articles about developing and validating factors affecting medication or treatment adherence [14, 20] and 19 qualitative studies about hypertension treatment experiences [21, 22] were used. At the end of literature review phase, 9 items were added to the item pool. Finally, at the end of item generation phase, the scale consists of 91 items. These items were categorized into 4 conceptual dimensions including (1) personal factors (53 items), (2) disease and treatment nature (16 items), (3) health system role (9 items), and (4) family-socio-cultural factors (13 items). A 4-point Likert type was used where 1 indicated “in my case is correct,” 2 indicated “in my case is somewhat correct,” 3 indicated “in my case is somewhat wrong,” and 4 indicated “in my case is wrong.” This kind of Likert scale was best fitted for all kinds of items. Higher scores indicated a lower level of confounding factors that may effect on hypertensive treatment adherence.

### 2.5. Sampling, Data Collection, and Analysis

We used five samples to conduct the preliminary validation study presented in this paper. A description of the samples, data collection, and analytic approaches taken are described next.

### 2.6. Sample 1

#### 2.6.1. Data Collection

The first sample collected face validity evidence from hypertensive patients to determine fit between the items comprising the scale and the concept of FEHTA (relevancy). In addition, the subject was asked to clarify items' difficulty and ambiguous. Participants consisted of 20 patients with hypertension in cardiovascular units of two educational hospitals and three cardiologist offices in Kerman, Iran. The participants were older than 18 years and take at least one antihypertensive agent. The sampling was convenience. The hypertensive patients, who were interested to participate in the study, were interviewed in regard with the scale items, and their suggestions about the items and scale were recorded; then, they were required to complete the scale according to five-point Likert scale (1 = not important, 2 = low important, 3 = somewhat important, 4 = important, and 5 = completely important). Data collection occurred in one interaction with each patient separately between October 2, 2014, and October 16, 2014.

#### 2.6.2. Data Analysis

Following interviews, the research team analyzed all comments recorded during the scale administration by content analysis. According to the results of content analysis, consensus on any changes to the scale occurred. Then, the item impact method was used to determine the importance of each item. To calculate item impact score, frequency of item importance multiply to the mean of item importance. According to the item impact method, if the item impact score is above 1.5, the item is important and should be maintained in the scale for further evaluation [23].

### 2.7. Sample 2

#### 2.7.1. Data Collection

The second sample was collected to evaluate content validity evidence from experts view. This phase consists of two steps. In the first step, fourteen experts were asked to write their comments about fitness, simplicity, and comprehensiveness of each item individually. In addition, they were asked to revise or edit the items that were not suitable enough. In the second step, to determine the necessity of each item (content validity ratio = CVR), the experts were asked to complete the scale according to the 3-point Likert scale (1 = not necessary, 2 = helpful but not necessary, and 3 = necessary). To determine the relevancy, simplicity, and clarity of each item and the scale (content validity index = CVI), the respondents were required to grade each item according to a 4-point Likert scale (for relevancy: 1 = not relevant, 2 = item needs major revision, 3 = relevant but needs minor revision, and 4 = very relevant; for simplicity: 1 = complex, 2 = item needs major revision, 3 = simple but needs minor revision, and 4 = very simple; for clarity: 1 = ambiguous, 2 = item needs major revision, 3 = clear but needs minor revision, and 4 = very clear). The experts consist of physicians (*n* = 6) and nursing faculties (*n* = 8) who were expert in research filed. A minimum of five experts are recommended for content validity assessment [24]. The scale was sent electronically to 8 experts. To collect the physicians' suggestions, the scale was printed and was offered to them in the educational hospitals in Kerman. This sampling lasted from October 27, 2014, to November 30, 2014.

#### 2.7.2. Data Analysis

The research team analyzed all experts' written comments by using content analysis. According to the results of content analysis, consensus on any changes to the scale occurred. To quantifying agreement on the scale content, content validity ratio (CVR) and content validity index (CVI) were used. Both indices allow for item-level assessment and are easy to interpret. The CVI also provides the scale-level assessment [24]. To calculate CVR for each item, the fallowing formula was used:

(1) $$\begin{array}{c} \text{CVR}=\frac{\text{nE}-\;N/2}{N/2}, \end{array}$$

where nE is the number of the experts who select the necessary option and *N* is the total number of experts.

According to the Lawshe table, when the total number of the experts is 14, the cut-point value would be 0.51 [25]. It means that each item that has CVR value less than 0.51 might be candidate for omission. To calculate CVI for each item (I-CVI), the number of experts giving a rating of either 3 or 4 is divided by the total number of experts. The I-CVI was calculated in regard to relevancy, simplicity, and clarity separately, and then the mean of these three issues was considered as I-CVI value of each item. The accepted standard in the literature for an I-CVI is 0.9. The S-CVI (the CVI score for the full FEHTA scale with all items) was calculated by taking the mean of the proportion of items that were rated either 3 or 4 across the 14 experts. A value of 0.80 or higher is considered acceptable [24].

### 2.8. Sample 3

#### 2.8.1. Data Collection

The third sample (pilot study) was collected to calculate internal consistency evidence of the FEHTA scale. Participants consisted of 50 patients with hypertension in cardiovascular units of two educational hospitals, three cardiologists, and two nephrologists' offices in Kerman, Iran. The participants were older than 18 years, had essential hypertension, and took at least one antihypertensive agent. The sampling was convenience. The hypertensive patients, who were interested to participate in the study, were interviewed. They were required to complete the scale according to 4-point Likert scale (1 = “in my case is correct,” 2 = “in my case is somewhat correct,” 3 = “in my case is somewhat wrong,” and 4 = “in my case is wrong”). Data collection lasted from December 10, 2014, to December 25, 2015.

#### 2.8.2. Data Analysis

Internal consistency refers to the extent to which items of the scale measure the same construct (i.e., homogeneity of the scale) and was assessed in this study by Cronbach's alpha coefficient. The coefficient can range from 0 to 1; a coefficient value more than 0.7 is considered acceptable [26].

### 2.9. Sample 4

#### 2.9.1. Data Collection

The fourth sample was collected to calculate construct validity, internal consistency, and practicability and acceptability of the FEHTA scale. The subjects consisted of hypertensive patients who were 18 or older and who had taken at least one antihypertensive medication. According to the literature, 5–10 subjects per item are enough for construct validity [27, 28]. Therefore, 325 hypertensive patients were selected by randomized multistage sampling. 160 subjects were taken from in-patient centers (13 hospital wards; cardiovascular, internal, and emergency wards), and 160 subjects were taken from out-patient centers (12 cardiologists and nephrologists' offices and the only subspecialty educational clinic). Each ward and office and the clinic were considered as a cluster, then three clusters of each stratum (out- and in-patient centers) were selected randomly, and the eligible participants were selected. Sociodemographic data, such as age, gender, marital status, educational and occupational status, duration of having hypertension, duration of taking antihypertensive drugs, and having other disease were collected. In addition, blood pressure was measured with an aneroid sphygmomanometer (ALPK2, Japan) using the average of two measurements taken five minutes apart. Systolic and diastolic blood pressures were obtained from the right arm of the subjects using standard procedure [29]. For illiterate individuals, interviews were used instead of the self-administered method. Data collection lasted from January 01, 2015, to February 25, 2015.

#### 2.9.2. Data Analysis

Exploratory factor analysis (EFA) was conducted to verify the factorial design of the FEHTA scale by using principal component analysis (PCA) with varimax rotation, [30]. The following criteria were used to determine the number of factors in the scales: eigenvalues >1, scree plots, and items with loadings of 0.4 or greater on any one factor [31].

Acceptability or practicability of the FEHTA scale was assessed by calculating missing values and the average time needed to fulfill the scale. Also, floor/ceiling effect was assessed. The amount of missing values and floor/ceiling effect should be less than 10% and 80%, respectively, in order that the scale obtains acceptability [32–34].

### 2.10. Sample 5

#### 2.10.1. Data Collection

The fifth sample was collected to determine test-retest reliability of the FEHTA scale. To do so, 50 patients, with hypertension in cardiovascular units of two educational hospitals, three cardiologists, and two nephrologists' offices in Kerman, completed the scale twice (at a two-week interval). The participants were older than 18 years, had essential hypertension, and took at least one antihypertensive agent. The sampling was convenience. The hypertensive patients, who were interested to participate in the study, were interviewed. Telephone contact has been used to collect data for the second time. Data collection lasted from March 01, 2015, to March 20, 2015.

#### 2.10.2. Data Analysis

We used the intraclass correlation coefficient (ICC) to assess repeatability of the FEHTA scale. To interpret the obtained coefficients, values above 0.7 were considered as excellent reliability [29]. In this study, all analyses were performed using SPSS version 19.0 (Statistical Package for the Social Sciences, SPSS, Chicago, IL, USA).

### 2.11. Ethical Consideration

Kerman University of Medical Sciences (KUMS) approved this project (ethic code = *K*/93/580). After approval of KUMS and coordination with research areas, we provided information for the subjects. The information addressed the objectives of the study and the confidentiality of the data, and the participants would be anonymous and were free to withdraw from the study at any time. Then, the informed consent was obtained verbally.

## 3. Results

### 3.1. Face Validity

A total of 20 hypertensive patients were recruited in the study to assessing face validity (a response rate of 100%). According to respondents' view, totally all items of the 91-item scale were simple and understandable; therefore, no change has been done in this phase. Twenty items had item impact scores below 1.5. The range of item impact scores among other items varied through 1.58 to 4.32. Based on item impact scores and importance of each item, the research team decided to omit seventeen items. Therefore, at the end of this phase, the scale consisted of 74 items.

### 3.2. Content Validity

A total of 14 (of 15) content validity surveys were returned (a response rate of 93.3%). According to experts' comments, eleven items were omitted or merged to other items due to “being not comprehensive or relevant” and “conceptual overlap,” respectively.  Table 1 summarizes the CVR and CVI scores. Nine items were below the accepted standard of CVR (<0.51) with a score range of −0.43 to 0.43. I-CVI scores of all items (between 0.81 and 1) were exceeded the accepted standard of >0.80. In addition, the S-CVI was 0.946. Finally, at the end of content validity phase, the FEHTA scale contained 53 items.

### 3.3. Pilot Study (Internal Consistency and Response Rate)

Three questionnaires were omitted due to missing values. The value of Cronbach's *α* for the FEHTA scale was 0.87. The FEHTA-item-total correlations ranged from −0.09 to 0.64. The item-total correlations were 0.20 or greater for 38 items of the 53-item FEHTA scale (Table 2). To improve Cronbach's *α* coefficient, three items that had negative item-total correlation were omitted and the internal consistency was recalculated. The alpha increased to 0.88. Therefore, at the end of this phase, the FEHTA scale consisted of 50 items.

### 3.4. Construct Validity

#### 3.4.1. Sociodemographic Characteristics

In total, 321 hypertensive patients were assessed. Four patients refused to participate in the study, so the response rate was more than 98%. The mean age of patients was 63.62 ± 7.4 years. 46.7% of the participants were men. Nearly 90% of participants were married and 50.3% were illiterate. 20% of participants were unemployed. The mean duration of having hypertension and taking antihypertensive drugs was 51.05 ± 23.52 months. 26.2% of participants had diabetes. 57.3% of the participants had other diseases (except for diabetes) (Table 3). The participants' responses to the FEHTA scale are summarized in Table 4. More than 50% of the participants believed that “29” and “47” items were correct in their own case. In addition, less than 5% of the participants believed that “3,” “5,” “30–32,” and “37–38” items were correct in their own case. The “31” item was deleted before calculating factor analysis because of being ambiguous and having 22 missing values.

#### 3.4.2. Exploratory Factor Analysis

To verify construct validity of the FEHTA scale, PCA with varimax rotation was used. In the first step, Bartlett and Kaiser–Meyer–Olkin (KMO) tests conducted to identify normal distribution of data and adequacy of sample size for EFA. The results of the Bartlett test were significant (*χ*^2^ = 7808.01; df = 1176, *P* < 0.001), and the KMO coefficient was 0.803 that exceeded the accepted standard of >0.7. In the second step, PCA with varimax rotation was conducted and 13 factors with an eigenvalue of >1 were retrieved. The total variance explained by these 13 factors was 68.51%.

According to scree plot that begins to level off after seventh factor, the EFA was conducted again by limiting PCA to a fixed number of 7-factor extraction and the result was assessed. The loading of 13 items (8, 9, 10, 11, 17, 18, 20, 28, 37, 38, 44, 47, and 49) was meaningful in related to their factors. Therefore, the EFA was conducted again by omitting these 13 items. In this step, KMO coefficient was 0.784 and the result of the Bartlett test was significant (*χ*^2^ = 5027.84; df = 630, *P* < 0.001). In the next step, PCA with varimax rotation was conducted and 9 factors with an eigenvalue of >1 were retrieved. The total variance explained by these 9 factors was 65.01%. In this step according to scree plot that begins to level off after fifth factor, the EFA was conducted again by limiting PCA to a fixed number of 5-factor extraction and the result was assessed. Three items [8, 31, 35] did not load in any factors. At this step, it seemed some items did not relate to their factors meaningfully. There are two ways to decide in these cases: (1) deleting the unrelated and not meaningful items and (2) considering the factor loading of the item in other factors though it be less than the interested amount of 0.4 [30]. Therefore, the factor loading of some items [1, 3, 7, 20, 26, 28, 36] in other factors was assessed. As the item no. 27 was not loaded in any factors except for the fourth factor and there was no relation between this item and other items loaded in the fourth factor, this item was deleted from the scale. Finally, at the end of EFA, the FEHTA scale consisted of 32 items that were placed in five meaningful factors (Table 5). In addition, correlation between FEHTA scale score and each dimension was between 0.04 and 0.66 and correlations of each dimension with other dimensions were between 0.03 and 0.35 (Table 6). Note that in order to calculate the factor analysis, missing values were replaced with means.

### 3.5. Acceptability and Practicability

The final FEHTA scale included 32 items and respondents were easily able to complete it, and the mean time for completing the scale was 11.15 ± 2.64 minutes (range: 5 to 20 minutes). In addition, only in 22.2% of patients, the time for completing the scale was more than 15 minutes. The missing values varied between 0 and 0.93% (mean = 0.27%). None of FEHTA scale items had floor/ceiling effect.

### 3.6. Reliability (Internal Consistency and Test-Retest)

To calculate reliability, the missing values were replaced with median. Cronbach's *α* for total sample size (*n* = 321) and ICC for a sample size of 50 patients were assessed. The value of Cronbach's *α* for whole of the scale was 0.71. The values of Cronbach's *α* of subscales were between 0.63 and 0.83 (Table 7). The results of test-retest of the two-week interval showed that the repeatability and stability of the scale were excellent (ICC: 0.89, CI: 0.81–0.94) (Table 7).

## 4. Discussion

Psychometric results of the scale “factors affecting treatment adherence of hypertensive patients” led to a scale with 32 items. This scale includes 5 dimensions that are beliefs and relationships (10 items), trust in treatment personnel and role of family (13 items), being busy and loneliness (4 items), medicinal side effects and indifference (3 items), and tendency to traditional medicine (2 items). The scale is scored based on Likert's four-point scale (it is true about me = 1, it is to some extent true about me = 2, it is to some extent false about me = 3, and it is false about me = 4). On this basis, the scores resulted from this scale will range from 32 to 128 and 5, 13, 21, 22, and 23 items were negative, so they reversed while scoring. The first psychometric stage of the scale was to study its face validity. In the present study, in addition to interview with patients and determine qualitatively face validity, the importance of the item was measured using effect factor of the item. For face validity, items that were given no acceptable score by respondents (items with scores lower than 1.5) that were not sufficiently comprehensive or overlapping items were omitted from the questionnaire. The most common and appropriate methods of determining the validity of a tool are to study content validity, criterion validity, and construct validity [35]. In the present study, after determining face validity of the scale, the second stage was to determine content validity. In fact, content validity means that how much the content of a tool can be representative of characteristics of the measurable construct [37]. If a tool is without content validity, study of its reliability will not be acceptable [24]. In the present study, in addition to qualitative study of views of specialists, the content validity index and content validity ratio have been used to quantify views of specialists. In the present study, the CVI-I index of all items of the scale was higher than 0.8 and CVI-S amount was higher than 0.9 showing appropriateness and acceptability of content validity. Appropriate content validity of a tool can be a starting point for doing other validity stages such as criterion and construct validities. Therefore, construct validity was studied in the next psychometric stage of the scale. Construct validity includes different methods such as cross cultural validity, structural validity, and hypothesis testing [38]. In the present study, factor structure of the scale was studied using exploratory factor analysis. Then, 5 factors were extracted by analysis of main components and varimax rotation and they explained 50.41% of total variance. The most common methods for extraction of factor in exploratory factor analysis are principle component analysis (PCA) and principal axis factoring (PAF). Although these methods are different [30, 39], Thompson stated that practical differences between these methods can be ignored in interpretation. Also, when reliability coefficient and the number of measuring variables are high, the difference between results of using different methods of factor extraction will be reduced [39]. Lorenzo-Seva added that only when the number of variables or communality is low, different ways of factor analysis and other methods of factor extraction such as PAF will have different results [36]. Therefore, in the present study, since the reliability coefficient of the scale was acceptable (0.7) and the number of variables was high (50), the scale was studied by both PCA and PAF. Then, concerning better interpretation and placement of items based on PCA, results related to this method was reported. Concerning the main aim of exploratory factor analysis (reaching the least factors with the highest variance), exploration of the proper number of factors is very important. Svarstad et al. suggested total variance more than 75% [40]. However, Henson and Roberts after studying variances explained in different studies (its average was 52%) doubted about reasonability of this amount in psychological studies [39]. However, in the present study, the amount of variance gained by 5 factors of the scale was higher than unexplored variance and the residual. Also, correlation coefficient between scores of each factor and total score was between 0.04 and 0.66 and these amounts were higher than correlation coefficients of each factor with other factors (0.03 and 0.30). It indicates that the scale has good construct validity [14]. Criterion validity is one of the most common validities [24]. In fact, criterion validity is correctness and suitableness of the score in reflecting a golden standard [35, 37]. Mokkink et al. stated that a golden standard that is used to measure criterion validity should be logical and acceptable [37] In literature review related to factors affecting the adherence, several tools have been psychometrically studied in different studies of which the most practical tools are “questionnaire of belief in medication” and “questionnaire of disease perception.” Criterion validity has not been reported in any studies related to psychometry of the “questionnaire of belief in medication” [41, 42]. Criterion validity has not been reported in some of studies related to psychometry of “questionnaire of disease perception” [16] and in some studies in which criterion validity has been reported, and many valid tools used in different researches have been applied as a golden standard [43]. On the other hand, Mokkink et al. stated that many authors considered mistakenly another tool (that is used extensively) as a golden standard [37]. Mokkink et al. stated in another study that there is no real golden standard about health questionnaires. They also noted whether criterion validity should be addressed in health questionnaires or not. This team finally stated that the only exception about criterion validity is that the short form of a tool is compared with the original long form. Therefore, the original form can be considered as a standard [35, 38]. Therefore, concerning lack of golden standard for factors affecting treatment adherence in hypertensive patients, dealing with criterion validity does not seem correctly for this scale. Thus, in the present study, criterion validity of the scale has not been evaluated. Reliability of the tool is as important as the validity of the tool [44]. Internal consistency shows the relation and correlation between items in a questionnaire [35, 37]. Alpha coefficient of the scale was 0.71. Reliability coefficient of 0.70 or higher is acceptable [26]. Amount of alpha is influenced by the number of items, item interrelatedness, and scale dimensions [44]. In the present study, amount of alpha for all subscales was higher than 0.70 except in the subscale “medicinal side effects and indifference.” Since amount of alpha is influenced by the number of items, obtaining amount of alpha lower than 0.63 for the subscale “medicinal side effects and laziness” (with three items) is justified. In fact, test-retest reliability is the amount of total variance and it associates with the real difference between patients. This aspect of reliability is reflected by using ICC or Cohen's Kappa [35]. Therefore, amount of test-retest reliability of the scale was estimated by calculation of ICC. Coefficient of test-retest reliability was very good (0.89) showing complementary evidence of the scale reliability. Practicability of the scale is very important for acceptance and usefulness of a scale. Ideally, a scale should be short, easily practical, and useful, and it should be so easy and understandable that result in no incorrect response [45]. Finally, the scale included 32 items and participants were easily able to complete it, and in average, it took 11 minutes (a proper time). Also, amount of missing values should be less than 10% and floor/ceiling effects are less than 80% in order that the acceptability of the scale is met [34]. Missing values were very low (0.27%) about the scale. Floor/ceiling effects concentrate on how to distribute the response to the scale. On the other hand, responding to one of high or low points in the Likert scale should not be higher than 0.80% of all responses. None of the items of the scale were well-accepted. The scale measures different dimensions such as beliefs and relationships, trust in treatment personnel and role of family, being busy and loneliness, medicinal side effects and indifference, and tendency towards traditional medicine. According to the conceptual framework offered by the World Health Organization in 2003, all factors affecting treatment adherence in patients with different chronic diseases are classified in five classes or dimensions that are factors related to the patient such as demographic features (age, gender, race, education, and marital status), sociopsychological factors (beliefs, motivation, and attitude), the relationship between doctor and patient, health literacy, patient knowledge, physical inabilities, consumption of tobacco or alcohol, and being unable to remember and experience of good adherence. Factors related to treatment are route of medicinal prescription, treatment complexity, treatment length, medicinal side effects, required behavioral changes, taste of medications, and requirements for drug storage. Factors related to the treatment system are limited access, waiting a long time, having problem in supplying prescription, and unhappy visits. Socioeconomic factors are inability to take a leave during work hours, cost, and income, social support, and factors related to disease which are symptoms, intensity, and seriousness of the disease [13]. Although Ma et al. recommended in their study that this conceptual framework can make formation of nonadherence structure possible [14], but concerning literature review, this conceptual framework has not been considered in methodological studies as a conceptual framework for factors affecting the adherence. Since this conceptual framework is not the disease specific, it seems that all areas mentioned for it do not have similar importance in patients with different diseases. In this regard, a study was done to determine factors affecting adherence in patients with different chronic diseases (hypertension, cardiac ischemic, cancer, hyperlipidemia, diabetics, and cardiac arrhythmia). Factors related to adherence were measured concerning WHO conceptual framework using questionnaires and different measuring tools. The most important factors that predict low adherence are affection to several diseases, affection to hypertension, number of pills taken daily, and complex treatment regime. Results indicate that all factors outlined in the conceptual framework offered by the WHO did not have the same importance in determination of low adherence and some of them had low and insignificant relation with low adherence [46]. Belief in medication and disease perception are of the most practical tools available for factors affecting treatment adherence [15, 16]. As it is clear, questionnaire of belief in medication concentrates on patient's belief and attitude towards medications in general and medications taken for a disease in particular [15]. The questionnaire of disease perception concentrates on different dimensions of patient's perception from his/her diseases. Disease perception means that understanding different aspects of disease and treatment includes the following things: symptoms attributed by the patient to the disease, personal beliefs in disease etiology, disease length perceived by the patient, expected effects and outcomes, and how a patient can control or recover from the disease [16]. The World Health Organization stated that the belief that only the patient is responsible for adhering treatment regime is misleading and indicates that there is no proper understanding on how other factors affect behavior of the person in adherence to treatment regime [7]. Another questionnaire studied psychometrically about medicinal nonadherence is the questionnaire of nonadherence causes (23 items). Authors addressed each item as a separated factor [9]. This approach makes decision on factors affecting adherence (mentioned in different studies) problematic. Therefore, concerning studies done in this regard and holistic approach of most studies, although dimensions of the scale are not as comprehensive as the conceptual framework offered by the WHO, this scale measures the role of important dimensions such as beliefs, relations, role of treatment personnel, role of family, being busy and loneliness, affection to medicinal side effects and tendency to traditional medicine on adherence in hypertensive patients. This scale provides a new tool for measuring factors affecting treatment adherence in hypertensive patients. In the present study, amount of validity and reliability indices may be influenced by several limitations available in the study. Although respondents are asked to answer the scale correctly, long primary scale (50 items) and oldness of patients (mean age of subjects was older than 60 years old) can have negative effect on correctness of scale completion thus on validity and reliability of the scale. However, despite of these limitations, validity and reliability indices were acceptable.

## 5. Conclusion

In recent years, treatment adherence in hypertensive patients has been highly paid attention to. Factors related to adherence to treatment regime in hypertensive patients are multiple and complex. Understanding and identification of common causes of nonadherence can help design and formulate proper strategies for settling obstacles. Different factors such as factors related to the patient, factors related to treatment, factors related to disease, characteristics of treatment team and system, and socioeconomic factors are effective on patient adherence to his/her treatment regime. No comprehensive tool has been designed to consider all these factors. The conceptual framework extracted from the study of qualitative content formed the basis of factors affecting treatment adherence in hypertensive patients, and a new scale was designed and studied psychometrically. Psychometric results of the scale indicate that this scale has a proper internal consistency and stability. Also, such scale has acceptable face validity. Content validity ratio and index of all items were acceptable. In addition, the total validity index of the scale was very good. Results of exploratory factor analysis indicate that this scale has five significant dimensions including beliefs and relations, trust in treatment personnel and role of family, being busy and loneliness, medicinal side effects, and laziness and tendency towards traditional medicine. The treatment team and nurses should prioritize the identification of the causes of nonadherence of patients to antihypertensive treatment regime because stable change of behaviors related to adherence in patients was not encouraging without identification and settlement of the causes of nonadherence. Application of the findings of the present research can be a proper step in promotion of caring hypertensive patients, and it is hoped that these findings are helpful in better and more effective care in clinic and they can be used in different practical and research fields.

## Figures and Tables

**Table 1:** Content validity ratio (CVR) and content validity index (CVI) scores of the FEHTA scale (*n* = 14).

No.	Items	CVR	CVI
1	I cannot trust in doctors	0.71	0.93
2	I visit the doctor only when I have a severe disease	0.43	0.95
3	Spending money and paying the doctor's visit is useless	0.86	0.98
4	Since my hypertension is genetic, treatment regime has no effect on its control	1	0.98
5	I believe that if I have no stress, my hypertension will be controlled and there is no need for treatment regime	1	1
6	Given hypertension compared to other diseases such as diabetes creates less stress for me	0.71	0.98
7	The hazards of chemical drugs such as blood pressure medications are over their benefits	0.71	0.98
8	I will become depended on antihypertensive medications if I take them regularly	0.86	0.93
9	Taking medication is enough to reduce hypertension, and there is no need to change life style	0.86	0.93
10	Since I do daily activities and I am housewife, exercising is not required	0.57	0.95
11	I do not concentrate on my treatment because I have no enough information about hypertension and its side effects	0.71	0.95
12	I cannot understand hypertension well	0.29	0.95
13	Blood pressure medications can cause interference in my life	0.57	0.95
14	If I affect a tolerable disease, it will not be necessary to visit the doctor	0.57	0.93
15	If the food is healthy in terms of material and spiritual can result physical and mental health	0.57	0.91
16	I will eat my favorite food even if it is not useful	0.29	0.86
17	I will use fast foods because they are delicious, available with suitable price	0.43	0.84
18	It is better to eat and die than not to eat and die	−0.43	0.81
19	If I consider my diet, the life will be difficult for me	0.71	1
20	I do not take my medications during travelling	0.43	0.98
21	I do not consider my diet or medicinal regime during travelling	0.71	0.98
22	I consider my diet at party or ceremonies	0.71	0.93
23	My health is always important for me	0.71	0.93
24	I do not like to consider my treatment regime although I am aware of its importance	0.86	1
25	I cannot consider my treatment regime well because I live lonely	0.71	0.93
26	I do not consider my diet, medicinal regime, or doing exercise due to indifference	1	0.91
27	I used to live mechanical life, so I do not want to exercise	0.71	0.93
28	I become very nervous because I am sensitive	0.14	1
29	I am affected by stress	0.71	1
30	I become very nervous due to improper relationship with my wife	0.57	1
31	I do not check my pressure due to fearing from blood pressure device	0.57	1
32	I follow my treatment regime because I fear from hypertension side effects	0.86	1
33	By observing the deaths of relatives following bleeding caused by high blood pressure, the more I follow my regimen	0.86	1
34	I rarely pay attention to my health and treatment regime due to difficulties in my life	0.86	1
35	When my medications are finished, I will buy them with delay	0.71	1
36	I will forget to take my medications if I am busy	0.43	1
37	I do not take my medications because nobody remembers me to take them	0.43	1
38	I will forget to take my medication when I am not at home	0.43	0.93
39	I eat fast foods such as pizza and sandwich due to lack of time and being very busy	0.86	0.88
40	I do not exercise as I am busy	0.86	0.91
41	I feel that I do not need medications	0.71	0.98
42	My body has depended on hypertension, and complete consideration of treatment regime has no effect on it	0.86	0.98
43	Because of the specific problem and not a sign, I do not see a doctor	0.57	0.98
44	I do not consider my treatment regime completely because I have no special problem with hypertension	0.57	0.91
45	I control my blood pressure after appearance of symptoms such as headache or creeping	0.86	0.95
46	I recognize my blood pressure without measuring with apparent clues	0.86	0.95
47	My blood pressure is not controlled even with taking medication and considering diet	0.71	0.95
48	My blood pressure is not reduced even with regular exercise	0.71	0.95
49	I do not visit the doctor because my hypertension has not responded to the treatment	0.86	0.93
50	My hypertension is not treated at all	0.71	0.88
51	It is difficult for me to consider medicinal regime because I have to take several medications	0.86	0.98
52	I take rarely my medications because of some problems such as coughing during consumption of hypertensive medications	0.86	0.91
53	I do not take my hypertensive medications because I have to take other drugs as well	0.71	0.95
54	I do not take my hypertensive medications because of frequent urination by taking such medications	0.86	0.93
55	Because of fear of drug interactions, sometimes I don't take my drugs	0.86	0.93
56	I sometimes do not take medications due to fearing from effect of them on sexual performance	0.86	0.93
57	Due to lack of money for providing drugs, I don't takemy drugs	0.86	0.98
58	Health and my blood pressure are followed by hospitals and doctors	0.71	0.95
59	Because the doctor or nurse had not had a good deal, I do not pay attention to their recommendations	1	0.95
60	Due to frequent explanation and education by doctors, I would follow their advice	0.86	0.95
61	If my doctor is skillful and experienced, I will listen to his advices	0.71	0.95
62	The doctor did not educate about my regime and medicines	0.71	0.95
63	My family insisted on me not to use foods that are not good for me	0.71	0.95
64	I do not follow my treatment regime when I am with my friends	0.57	0.95
65	I listen to my friends' advice about my hypertensive medicinal regime without consulting with the doctor	0.86	0.88
66	I use opium to reduce my hypertension due to my friends' advice	0.71	0.88
67	I eat foods with cool temper to reduce my hypertension	0.57	0.88
68	When I eat fatty foods, I use lemon juice to reduce the fatty effect	0.71	0.88
69	I use regularly vegetative drugs instead of chemical ones	0.86	0.93
70	I follow educational programs about hypertension in media	0.71	0.95
71	My wife and children insisted on me to consider my diet and treatment regime	0.57	0.93
72	My life is without stress	0.86	0.95
73	Because in family members, we have doctors and nurses, I do not go to the doctor	0.86	0.95
74	All my family members pay attention to their health	0.71	0.95

Acceptable CVR scores >0.51 and acceptable CVI scores >0.8.

**Table 2:** Internal consistency of factors affecting the hypertensive treatment adherence scale (pilot study, *n* = 47).

No.	Items	Cronbach's alpha if item was deleted	Corrected item-total correlation
1	Since my hypertension is genetic, treatment regime has no effect on its control	0.87	0.27
2	I believe that if I have no stress, my hypertension will be controlled and there is no need for treatment regime	0.86	0.61
3	I will become depended on antihypertensive medications if I take them regularly	0.87	0.38
4	Taking medication is enough to reduce hypertension and there is no need to change life style	0.87	0.19
5	I feel that I do not need medications	0.87	−0.02
6	I cannot trust in doctors	0.87	0.49
7	Spending money and paying the doctor's visit is useless	0.87	0.27
8	Since I do daily activities and I am housewife, exercising is not required	0.87	0.50
9	I do not concentrate on my treatment because I have no enough information about hypertension and its side effects	0.87	0.40
10	If I affect a tolerable disease, it will not be necessary to visit the doctor	0.87	0.19
11	If I consider my diet, the life will be difficult for me	0.86	0.56
12	I do not consider my diet or medicinal regime during travelling	0.87	0.42
13	I consider my diet at party or ceremonies	0.87	0.16
14	My health is always important for me	0.87	0.42
15	I do not like to consider my treatment regime although I am aware of its importance	0.87	0.10
16	When I'm alone, I can't follow my regimen well	0.87	0.31
17	I do not consider my diet, medicinal regime, or doing exercise due to indifference	0.87	0.44
18	I am affected by stress	0.87	0.16
19	I become very nervous due to improper relationship with my wife	0.87	0.26
20	I do not check my pressure due to fearing from blood pressure device	0.87	0.49
21	I follow my treatment regime because I fear from hypertension side effects	0.87	0.12
22	I rarely pay attention to my health and treatment regime due to difficulties in my life	0.86	0.55
23	When my medications are finished, I will buy them with delay	0.87	0.53
24	I eat fast foods such as pizza and sandwich due to lack of time and being very busy	0.87	0.24
25	I do not exercise as I am busy	0.87	0.18
26	I do not take my hypertensive medications because I have to take other drugs as well	0.86	0.54
27	My body has depended on hypertension, and complete consideration of treatment regime has no effect on it	0.87	0.24
28	I do not consider my treatment regime completely because I have no special problem with hypertension	0.87	0.40
29	I control my blood pressure after appearance of symptoms such as headache or creeping	0.87	0.47
30	I recognize my blood pressure without measuring with apparent clues	0.87	0.46
31	My blood pressure is not controlled even with taking medication and considering diet	0.87	0.42
32	My blood pressure is not reduced even with regular exercise	0.87	0.31
33	I do not visit the doctor because my hypertension has not responded to the treatment	0.86	0.64
34	My hypertension is not treated at all	0.87	0.41
35	It is difficult for me to consider medicinal regime because I have to take several medications	0.87	0.30
36	I take rarely my medications because of some problems such as coughing during consumption of hypertensive medications	0.87	0.56
37	I do not take my hypertensive medications because of frequent urination by taking such medications	0.87	0.42
38	I sometimes do not take medications due to fearing from effect of them on sexual performance	0.87	0.26
39	Due to lack of money for providing drugs, I donot takemy drugs	0.87	0.26
40	Because the doctor or nurse had not had a good deal, I do not pay attention to their recommendations	0.87	0.47
41	Due to frequent explanation and education by doctors, I would follow their advice	0.87	0.31
42	If my doctor is skillful and experienced, I will listen to his advices	0.87	0.27
43	My family insisted on me not to use foods that are not good for me	0.87	0.41
44	I do not follow my treatment regime when I am with my friends	0.87	0.43
45	I listen to my friends' advice about my hypertensive medicinal regime without consulting with the doctor	0.88	−0.02
46	I use opium to reduce my hypertension due to my friends' advice	0.87	0.28
47	I eat foods with cool temper to reduce my hypertension	0.87	0.11
48	When I eat fatty foods, I use lemon juice to reduce the fatty effect	0.87	0.11
49	I use regularly vegetative drugs instead of chemical ones	0.88	−0.09
50	I follow educational programs about hypertension in media	0.87	0.17
51	My wife and children insisted on me to consider my diet and treatment regime	0.87	0.16
52	My life is without stress	0.87	0.27
53	All my family members pay attention to their health	0.87	0.19

**Table 3:** Description of the study sample (*n* = 321).

*Quantitative variables*		Mean (SD)
Age (yr)		63.62 ± 7.4
Duration of having hypertension (mo)		51.05 ± 23.52
Duration of treatment for hypertension (mo)		51.05 ± 23.52
Systolic blood pressure (mmHg)		135.69 ± 8.22
Diastolic blood pressure (mmHg)		87.09 ± 6.75

*Qualitative variables*		Frequency (%^*∗*^)
Gender	Female	170 (53.3)
	Male	149 (46.7)

Marital status	Single	4 (1.3)
	Married	280 (87.8)
	Divorced	4 (1.3)
	Widow(er)	31 (9.6)

Education status	Illiterate	160 (50.3)
	Under diploma	46 (14.5)
	Diploma	77 (24.2)
	Bachelor's degree or above	35 (11)

Occupation	Unemployed	64 (20)
	Employed	103 (32.2)
	Pensioner	56 (17.5)
	Housewife	97 (30.3)

Antihypertensive drugs	One drug	30 (9.4)
	Two drugs	227 (70.7)
	Three drugs or more	64 (19.9)

Having diabetes mellitus	Yes	84 (26.2)
	No	237 (73.8)

Having other diseases except for diabetes	Yes	184 (57.3)
	No	137 (42.7)

^*∗*^Valid percent.

**Table 4:** Distribution of the responses to the factors affecting hypertensive treatment adherence scale (*n* = 321).

No.	Item	Missing (*n*)	Response, *n* (%^*∗*^)			
			In my case is correct	In my case is somewhat correct	In my case is somewhat wrong	In my case is wrong
1	Since my hypertension is genetic, treatment regime has no effect on its control	0	26 (8.1)	51 (15.9)	159 (49.5)	85 (26.5)
2	I believe that if I have no stress, my hypertension will be controlled and there is no need for treatment regime	0	81 (25.2)	93 (29)	90 (28)	57 (17.8)
3	I will become depended on antihypertensive medications if I take them regularly	2	15 (4.7)	61 (19.1)	144 (45.1)	99 (31)
4	Taking medication is enough to reduce hypertension and there is no need to change life style	2	64 (20.1)	86 (27)	106 (33.2)	63 (19.7)
5	I cannot trust in doctors	0	15 (4.7)	55 (17.1)	118 (36.8)	133 (41.4)
6	Spending money and paying the doctor's visit is useless	3	28 (8.8)	81 (25.5)	86 (27)	123 (38.7)
7	Since I do daily activities and I am housewife, exercising is not required	1	75 (23.4)	156 (48.8)	58 (18.1)	31 (9.7)
8	I do not concentrate on my treatment because I have no enough information about hypertension and its side effects	0	37 (11.5)	69 (21.5)	119 (37.1)	96 (29.9)
9	If I affect a tolerable disease, it will not be necessary to visit the doctor	0	15 (4.7)	7 (27.1)	126 (39.3)	93 (29)
10	If I consider my diet, the life will be difficult for me	0	64 (19.9)	148 (46.1)	59 (18.4)	50 (15.6)
11	I do not take my medications during travelling	2	61 (19.1)	67 (21)	135 (42.3)	56 (17.6)
12	I consider my diet at party or ceremonies	1	79 (24.7)	111 (34.7)	73 (22.8)	57 (17.8)
13	My health is always important for me	1	156 (48.8)	121 (37.8)	24 (7.5)	19 (5.9)
14	I do not like to consider my treatment regime although I am aware of its importance	1	100 (31.3)	102 (31.9)	89 (27.8)	29 (9.1)
15	When I am alone, I cannot follow my regimen well	1	52 (16.3)	68 (21.3)	127 (39.7)	73 (22.8)
16	I do not consider my diet, medicinal regime, or doing exercise due to indifference	2	59 (18.5)	168 (52.7)	73 (22.9)	19 (6)
17	I am affected by stress	2	66 (20.7)	103 (32.3)	104 (32.6)	46 (14.4)
18	I become very nervous due to improper relationship with my wife	4	68 (21.5)	101 (31.9)	81 (25.6)	67 (21.1)
19	I do not check my pressure due to fearing from blood pressure device	3	46 (14.5)	32 (10.1)	106 (33.3)	134 (42.1)
20	I follow my treatment regime because I fear from hypertension side effects	1	87 (27.2)	92 (28.8)	72 (22.5)	69 (21.6)
21	I rarely pay attention to my health and treatment regime due to difficulties in my life	1	88 (27.5)	100 (31.3)	77 (24.1)	55 (17.2)
22	When my medications are finished, I will buy them with delay	0	36 (11.2)	78 (24.3)	112 (34.9)	95 (29.6)
23	I eat fast foods such as pizza and sandwich due to lack of time and being very busy	0	52 (16.2)	50 (15.6)	131 (40.8)	88 (27.4)
24	I do not exercise as I am busy	0	50 (15.6)	70 (21.8)	132 (41.1)	69 (21.5)
25	I do not take my hypertensive medications because I have to take other drugs as well	0	45 (14)	87 (27.1)	121 (37.7)	68 (21.2)
26	My body has depended on hypertension, and complete consideration of treatment regime has no effect on it	0	32 (10)	109 (34)	96 (29.9)	84 (26.2)
27	I do not consider my treatment regime completely because I have no special problem with hypertension	1	63 (19.7)	135 (42.2)	72 (22.5)	50 (15.6)
28	I control my blood pressure after appearance of symptoms such as headache or creeping	0	124 (38.6)	138 (43)	50 (15.6)	9 (2.8)
29	I recognize my blood pressure without measuring with apparent clues	1	184 (57.5)	119 (37.2)	14 (4.4)	3 (0.9)
30	My blood pressure is not controlled even with taking medication and considering diet	0	15 (4.7)	23 (7.2)	154 (48)	129 (40.2)
31	My blood pressure is not reduced even with regular exercise	22	6 (2)	32 (10.7)	139 (46.5)	122 (40.8)
32	I do not visit the doctor because my hypertension has not responded to the treatment	1	2 (0.6)	27 (8.4)	135 (42.2)	156 (48.8)
33	My hypertension is not treated at all	3	32 (10.1)	87 (27.4)	120 (37.7)	79 (24.8)
34	It is difficult for me to consider medicinal regime because I have to take several medications	1	104 (32.5)	147 (45.9)	49 (15.3)	20 (6.3)
35	I take rarely my medications because of some problems such as coughing during consumption of hypertensive medications	0	98 (30.5)	135 (42.1)	65 (20.2)	23 (7.2)
36	I do not take my hypertensive medications because of frequent urination by taking such medications	0	96 (29.9)	141 (43.9)	63 (19.6)	21 (6.5)
37	I sometimes do not take medications due to fearing from effect of them on sexual performance	1	6 (1.9)	6 (1.9)	170 (53.1)	138 (43.1)
38	Due to lack of money for providing drugs, I donot takemy drugs	0	6 (1.9)	11 (3.4)	138 (43)	166 (51.7)
39	Because the doctor or nurse had not had a good deal, I do not pay attention to their recommendations	0	27 (8.4)	44 (13.7)	144 (44.9)	106 (33)
40	Due to frequent explanation and education by doctors, I would follow their advice	1	97 (30.3)	100 (31.3)	71 (22.2)	52 (16.3)
41	If my doctor is skillful and experienced, I will listen to his advices	3	133 (41.8)	145 (45.6)	29 (9.1)	11 (3.5)
42	My family insisted on me not to use foods that are not good for me	0	44 (13.7)	102 (31.8)	126 (39.3)	49 (15.3)
43	I do not follow my treatment regime when I am with my friends	1	99 (30.9)	80 (25)	86 (26.9)	55 (17.2)
44	I use opium to reduce my hypertension due to my friends' advice	1	24 (7.5)	84 (26.3)	139 (43.4)	73 (22.8)
45	I eat foods with cool temper to reduce my hypertension	1	115 (35.9)	160 (50)	26 (8.1)	19 (5.9)
46	When I eat fatty foods, I use lemon juice to reduce the fatty effect	0	139 (43.3)	146 (45.5)	24 (7.5)	12 (3.7)
47	I follow educational programs about hypertension in media	0	161 (50.2)	131 (40.8)	13 (4)	16 (5)
48	My wife and children insisted on me to consider my diet and treatment regime	3	118 (37.1)	150 (47.2)	38 (11.9)	12 (3.8)
49	My life is without stress	1	60 (18.8)	83 (25.9)	128 (40)	49 (15.3)
50	All my family members pay attention to their health	0	143 (44.5)	157 (48.9)	17 (5.3)	4 (1.2)

^*∗*^Valid percent.

**Table 5:** Rotated factor matrix: the factors affecting the hypertensive treatment adherence scale.

No.	Items	Factor 1	Factor 2	Factor 3	Factor 4	Factor 5
		Beliefs and relations	Trust on health providers and family role	Busy and being alone	Medication side effects	Traditional medicine
4	Taking medication is enough to reduce hypertension, and there is no need to change life style	0.74				
2	I believe that if I have no stress, my hypertension will be controlled and there is no need for treatment regime	0.69				
26	My body has depended on hypertension, and complete consideration of treatment regime has no effect on it	0.68				
33	My hypertension is not treated at all	0.64				
40	Due to frequent explanation and education by doctors, I would follow their advice	0.73				
42	My family insisted on me not to use foods that are not good for me	0.62				
14	I do not like to consider my treatment regime although I am aware of its importance	0.66				
43	I do not follow my treatment regime when I am with my friends	0.44		0.50		
21	I rarely pay attention to my health and treatment regime due to difficulties in my life	0.60				
25	I do not take my hypertensive medications because I have to take other drugs as well	0.42				
5	I cannot trust in doctors		0.58			
39	Because the doctor or nurse had not had a good deal, I do not pay attention to their recommendations		0.36	0.30	0.54	
41	If my doctor is skillful and experienced, I will listen to his advices		0.51			
6	Spending money and paying the doctor's visit is useless		0.34	0.36		0.50
1	Since my hypertension is genetic, treatment regime has no effect on its control		0.48			0.49
32	I do not visit the doctor because my hypertension has not responded to the treatment		0.65			
3	I will become depended on antihypertensive medications if I take them regularly		0.26	0.49		
30	My blood pressure is not controlled even with taking medication and considering diet		0.65			
19	I do not check my pressure due to fearing from blood pressure device		0.29	0.47	0.34	
22	When my medications are finished, I will buy them with delay		0.41			
50	All my family members pay attention to their health		0.40			
48	My wife and children insisted on me to consider my diet and treatment regime		0.56			
13	My health is always important for me		0.51			
12	I consider my diet at party or ceremonies			0.74		
24	I do not exercise as I am busy			0.66		
23	I eat fast foods such as pizza and sandwich due to lack of time and being very busy			0.61		
15	When I am alone, I cannot follow my regimen well			0.77		
35	I take rarely my medications because of some problems such as coughing during consumption of hypertensive medications				0.73	
36	I do not take my hypertensive medications because of frequent urination by taking such medications				0.65	
16	I do not consider my diet, medicinal regime, or doing exercise due to indifference				0.49	
45	I eat foods with cool temper to reduce my hypertension					0.74
46	When I eat fatty foods, I use lemon juice to reduce the fatty effect					0.67
7	Since I do daily activities and I am housewife, exercising is not required	−0.41				
34	It is difficult for me to consider medicinal regime because I have to take several medications	−0.62				
27	I do not consider my treatment regime completely because I have no special problem with hypertension			0.58		
29	I recognize my blood pressure without measuring with apparent clues	—	—	—	—	—
Eigenvalue		5.38	4.67	3.24	2.68	2.16
Explained variance (%)		14.95	12.98	9.02	7.45	6.02

**Table 6:** Correlations between the factors affecting the hypertensive treatment adherence scale score and its subscales.

Factors	Factor 1	Factor 2	Factor 3	Factor 4	Factor 5	Total
Factor 1	1					
Factor 2	−0.06	1				
Factor 3	−0.10	0.35^*∗*^	1			
Factor 4	0.06	−0.20^*∗*^	−0.28^*∗*^	1		
Factor 5	−0.11	0.03	0.14^*∗*^	0.03	1	
Total	0.61^*∗*^	0.66^*∗*^	0.44^*∗*^	0.04	0.12^*∗*^	1

^*∗*^ *P* < 0.05.

**Table 7:** Internal consistency of factors affecting the hypertensive treatment adherence scale and intraclass correlation.

No.	Items	Cronbach's alpha if item was deleted (*n* = 321)	Corrected item-total correlation	ICC (confidence interval) (*n* = 50)
4	Taking medication is enough to reduce hypertension, and there is no need to change life style	0.70	0.29	0.78 (0.64–0.87)
2	I believe that if I have no stress, my hypertension will be controlled and there is no need for treatment regime	0.70	0.31	0.73 (0.58–0.84)
26	My body has depended on hypertension, and complete consideration of treatment regime has no effect on it	0.71	0.10	0.82 (0.70–0.89)
33	My hypertension is not treated at all	0.71	0.16	0.82 (0.70–0.89)
40	Due to frequent explanation and education by doctors, I would follow their advice	0.70	0.28	0.68 (0.50–0.80)
42	My family insisted on me not to use foods that are not good for me	0.69	0.40	0.87 (0.79–0.93)
14	I do not like to consider my treatment regime although I am aware of its importance	0.69	0.47	0.85 (0.75–0.91)
43	I do not follow my treatment regime when I am with my friends	0.68	0.50	0.84 (0.74–0.91)
21	I rarely pay attention to my health and treatment regime due to difficulties in my life	0.70	0.26	0.87 (0.79–0.093)
25	I do not take my hypertensive medications because I have to take other drugs as well	0.71	0.09	0.80 (0.67–0.88)

*Beliefs and relations*		0.83		0.94 (0.90–0.97)
5	I cannot trust in doctors	0.70	0.22	0.70 (0.52–0.82)
39	Because the doctor or nurse had not had a good deal, I do not pay attention to their recommendations	0.70	0.24	0.86 (0.76–0.92)
41	If my doctor is skillful and experienced, I will listen to his advices	0.70	0.26	0.39 (0.12–0.60)
6	Spending money and paying the doctor's visit is useless	0.70	0.28	0.85 (0.75–0.91)
1	Since my hypertension is genetic, treatment regime has no effect on its control	0.70	0.28	0.80 (0.67–0.88)
32	I do not visit the doctor because my hypertension has not responded to the treatment	0.70	0.32	0.59 (0.38–0.74)
3	I will become depended on antihypertensive medications if I take them regularly	0.71	0.18	0.71 (0.53–0.82)
30	My blood pressure is not controlled even with taking medication and considering diet	0.70	0.22	0.74 (0.58–0.84)
19	I do not check my pressure due to fearing from blood pressure device	0.70	0.30	0.91 (0.85–0.95)
22	When my medications are finished, I will buy them with delay	0.70	0.28	0.81 (0.69–0.89)
50	All my family members pay attention to their health	0.70	0.38	0.30 (0.03–0.54)
48	My wife and children insisted on me to consider my diet and treatment regime	0.70	0.24	0.56 (0.33–0.72)
13	My health is always important for me	0.70	0.28	0.41 (0.15–0.62)

*Trust on health providers and family role*		0.78		0.87 (0.78–0.92)
12	I consider my diet at party or ceremonies	0.70	0.31	0.84 (0.74–0.91)
24	I do not exercise as I am busy	0.72	0.04	0.80 (0.66–0.88)
23	I eat fast foods such as pizza and sandwich due to lack of time and being very busy	0.71	0.11	0.76 (0.61–0.86)
15	When I am alone, I cannot follow my regimen well	0.69	0.44	0.90 (0.82–0.94)

*Busy and being alone*		0.73		0.89 (0.81–0.94)
35	I take rarely my medications because of some problems such as coughing during consumption of hypertensive medications	0.72	−0.07	0.86 (0.76–0.92)
36	I do not take my hypertensive medications because of frequent urination by taking such medications	0.72	−0.09	0.81 (0.69–0.89)
16	I do not consider my diet, medicinal regime, or doing exercise due to indifference	0.72	−0.02	0.60 (0.39–0.75)
*Medication side effects*		0.63		0.87 (0.78–0.92)
45	I eat foods with cool temper to reduce my hypertension	0.72	0.03	0.66 (0.47–0.79)
46	When I eat fatty foods, I use lemon juice to reduce the fatty effect	0.71	0.06	0.84 (0.74–0.91)

*Traditional medicine*		0.75		0.82 (0.70–0.90)

## Data Availability

The datasets used for the current study are available from the corresponding author upon request.
